# Comparative exploration of mammalian deafness gene homologues in the *Drosophila* auditory organ shows genetic correlation between insect and vertebrate hearing

**DOI:** 10.1371/journal.pone.0297846

**Published:** 2024-02-27

**Authors:** Daniel C. Sutton, Jonathan C. Andrews, Dylan M. Dolezal, Ye Jin Park, Hongjie Li, Daniel F. Eberl, Shinya Yamamoto, Andrew K. Groves

**Affiliations:** 1 Graduate Program in Genetics & Genomics, Baylor College of Medicine, Houston, Texas, United States of America; 2 Department of Molecular and Human Genetics, Baylor College of Medicine, Houston, Texas, United States of America; 3 Jan and Dan Duncan Neurological Research Institute, Texas Children’s Hospital, Houston, Texas, United States of America; 4 Department of Biology, University of Iowa, Iowa City, Iowa, United States of America; 5 Graduate Program in Development, Disease Models & Therapeutics, Baylor College of Medicine, Houston, Texas, United States of America; 6 Huffington Center on Aging, One Baylor Plaza, Houston, Texas, United States of America; 7 Department of Neuroscience, Baylor College of Medicine, Houston, Texas, United States of America; Shaheed Rajaei Hospital: Rajaie Cardiovascular Medical and Research Center, ISLAMIC REPUBLIC OF IRAN

## Abstract

Johnston’s organ, the *Drosophila* auditory organ, is anatomically very different from the mammalian organ of Corti. However, recent evidence indicates significant cellular and molecular similarities exist between vertebrate and invertebrate hearing, suggesting that *Drosophila* may be a useful platform to determine the function of the many mammalian deafness genes whose underlying biological mechanisms are poorly characterized. Our goal was a comprehensive screen of all known orthologues of mammalian deafness genes in the fruit fly to better understand conservation of hearing mechanisms between the insect and the fly and ultimately gain insight into human hereditary deafness. We used bioinformatic comparisons to screen previously reported human and mouse deafness genes and found that 156 of them have orthologues in *Drosophila melanogaster*. We used fluorescent imaging of T2A-GAL4 gene trap and GFP or YFP fluorescent protein trap lines for 54 of the *Drosophila* genes and found 38 to be expressed in different cell types in Johnston’s organ. We phenotypically characterized the function of strong loss-of-function mutants in three genes expressed in Johnston’s organ (*Cad99C*, *Msp-300*, and *Koi*) using a courtship assay and electrophysiological recordings of sound-evoked potentials. *Cad99C* and *Koi* were found to have significant courtship defects. However, when we tested these genes for electrophysiological defects in hearing response, we did not see a significant difference suggesting the courtship defects were not caused by hearing deficiencies. Furthermore, we used a UAS/RNAi approach to test the function of seven genes and found two additional genes, *CG5921* and *Myo10a*, that gave a statistically significant delay in courtship but not in sound-evoked potentials. Our results suggest that many mammalian deafness genes have *Drosophila* homologues expressed in the Johnston’s organ, but that their requirement for hearing may not necessarily be the same as in mammals.

## Introduction

Three out of every 1,000 individuals are born with a detectable level of hearing loss in one or both ears [1, 2]. About half of these cases are due to inherited mutations [3]. Historically, genetic screening of hearing-impaired families has been used to identify genes necessary for normal hearing function. In addition, forward genetic screens and comprehensive targeted gene inactivation projects in mice have identified many new candidate genes required for hearing function that have yet to be identified in human populations [4–8]. While this method has proven to be effective in identifying large numbers of deafness genes, the molecular functions of many of these genes, and the mechanisms by which pathogenic variants in these genes cause hearing loss has remained largely unexplored.

**Fig 1:**
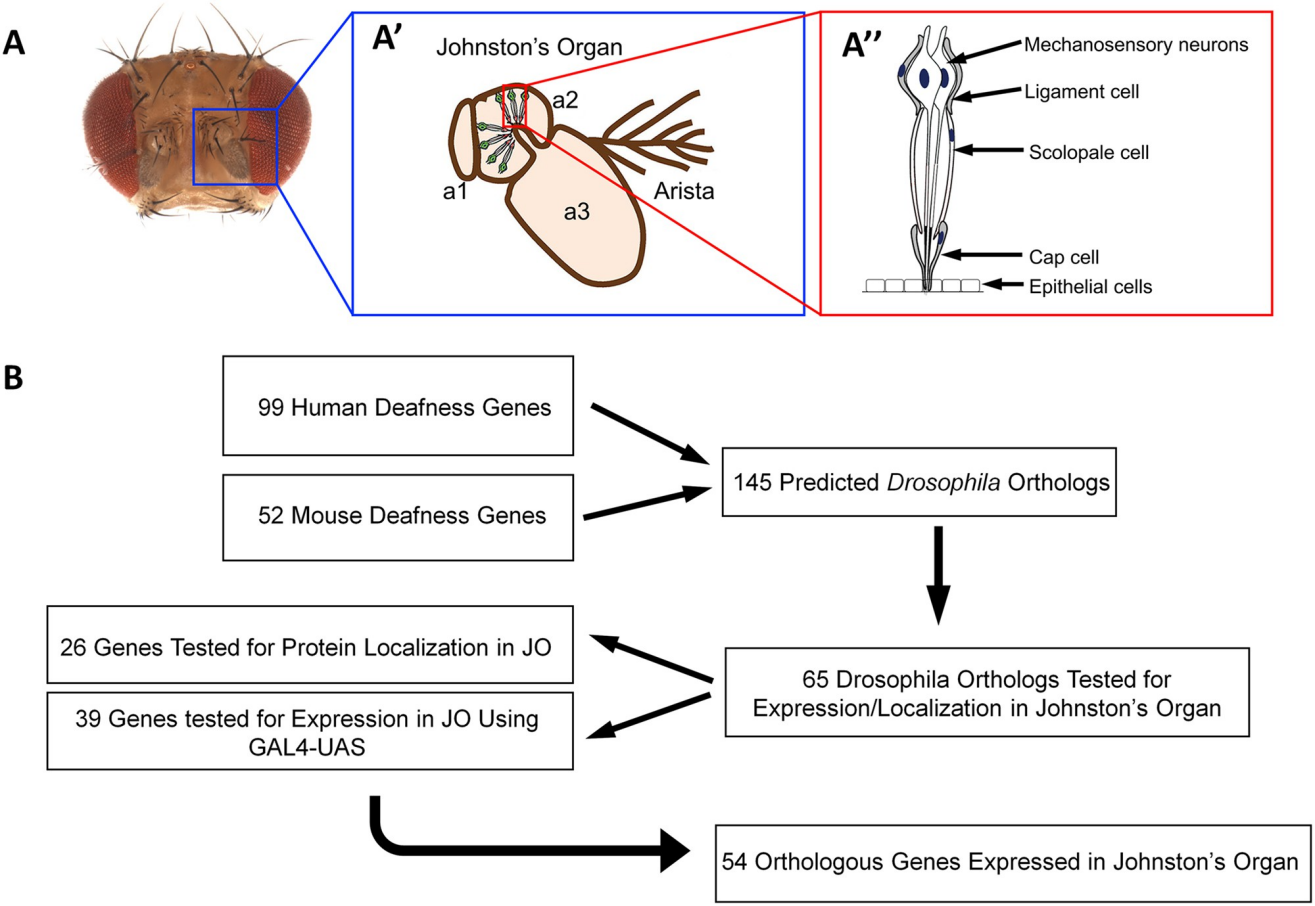
(A) Picture of an adult Drosophila head with red arrows denoting the second antennal segment containing Johnston’s organ (A’) a cartoon representing the fly antenna showing individual scolopidia within the second antennal segment (A”), a cartoon showing the individual cell types of the scolopidia. (B) A diagrammatic description of the workflow of the current project. A pool of human and mouse deafness genes were sorted through an orthologue prediction software to identify which mammalian genes had orthologues within Drosophila. Orthologous genes were prioritized for testing based on availability of reagents for gene expression and protein localization studies. Conservation of deafness genes was determined by which genes were expressed or localized within Johnston’s organ.

The fruit fly, Drosophila melanogaster, provides the ability to functionally validate genes more rapidly compared to mammalian models such as the mouse [9–11]. Drosophila and humans have a high degree of functional conservation in signaling pathways and disease-causing genes [12–14]. The Undiagnosed Disease Network (UDN), a large collaborative project with the aim to improve human health by identifying the molecular underpinnings of genetic disorders and develop novel therapies, has shown that Drosophila can be used effectively to study human disease [15–17]. Humans and flies also detect sound and gravity with specialized mechanosensory organs that are evolutionarily correlated [18, 19]. Mammals detect sound with mechanosensitive hair cells within the organ of Corti of the inner ear [20]. Actin-rich projections, known as stereovilli or stereocilia, protrude from the apical surface of hair cells which, when deflected by sound waves entering through the ear canal, lead to the opening of mechanically gated ion channels, development of a receptor potential, and synaptic transmission to activate neurons in the auditory pathway [21]. The hearing organ of Drosophila, Johnston’s organ (JO), is located within the second segment of the antenna and is comprised of several hundred functional units called scolopidia [18, 22]. These scolopidia contain one to three mechanosensory neurons surrounded by an actin-rich scolopale cell and are anchored to each side of the second antennal segment cuticle by ligament and cap cells (Fig 1A, 1A’ and 1A”). Rotation of the second antennal joint caused by near-field sound displacement, gravity, or air flow applies force to the ciliary dendrites of the mechanosensory neurons, leading to their depolarization [18, 22]. While the anatomical structure of fly and mammalian hearing organs is very different, they share a surprising degree of functional and molecular properties [22–24].

Taking advantage of the similarities between mammalian and fly hearing, we previously used the Drosophila to screen for genes relevant to human hereditary deafness [24]. We showed that genes involved in two forms of syndromic hearing loss that have been well-characterized in mammals, Usher syndrome and MYH9-related disorders, also contribute to in hearing in Drosophila [24]. An E3 ubiquitin ligase, Ubr3, genetically and physically interacts with the fly orthologues of the Usher syndrome causing human genes PCDH15 (Protocadherin 15, also known as USH1F), MYO7A (Myosin VIIA, also known as USH1B), and USH1G [also known as Sans (scaffold protein containing ankyrin repeats and SAM domain)]. Ubr3 also regulates Zipper, a Myosin II-family motor protein that is linked to MYH9-related diseases in human. This was the first evidence of two seemingly unrelated mammalian deafness disorders being linked using Drosophila as a model system to study the molecular characteristics of homologous genes.

In the present study, we aimed to expand the catalogue of genes known to be evolutionarily conserved between flies and humans that are involved in hearing. We performed an expression-based screen to assess gene expression and protein localization of mammalian deafness gene homologues in the fly. Starting with a list of deafness genes identified in humans and mice, we searched for genes that are conserved in the fruit fly and identified which of these orthologue candidates are expressed in Johnston’s organ using fluorescent gene and protein trap reporters. To assess whether we can identify novel genes involved in fly hearing, we tested whether three mammalian deafness genes–PCDH15 (fly: Cad99C), SUN1 (fly: koi), and SYNE4 (fly: Msp300)–were functionally conserved based on a Drosophila mating behavior assay which is dependent on hearing and through electrophysiological recordings of sound evoked potentials (SEPs). We found that mutant alleles of koi and Cad99C showed statistically significant effects on courtship behavior but not for SEPs. We expanded the results of our screen using a RNAi-based gene knockdown approach by combining UAS-RNAi with the T2A-GAL4 system to produce partial loss-of-function alleles that would be viable in adulthood. Using this approach, we found two genes that had significant mating defects–homologs of human MYO15 (fly: Myo10A) and PCDH15 (fly: Cad99C) but not in SEP’s. Our study reveals many mammalian deafness genes have conserved fly homologues that are expressed within the Johnston’s organ. We suggest that using the broad genetic toolkits developed in Drosophila to study human hereditary deafness can allow for functionally conserved deafness genes to provide novel mechanistic insights into genes required for normal hair cell function in vertebrates.

## Materials and methods

### Identification of candidate Drosophila orthologues of human and mouse deafness genes

A list of human and mouse deafness genes was compiled from an web-based database and published literature (https://hereditaryhearingloss.org/ accessed 2020, https://www.mousephenotype.org/, [4]; S1 Table). Orthologue candidates for human and mouse deafness genes were obtained using the DRSC Integrative Orthologue Prediction Tool (DIOPT version 7—[13]). Fly genes with the highest ranked weighted scores were considered the most likely orthologue candidate genes. Human and mouse deafness genes with multiple orthologue candidates sharing the highest DIOPT scores were also prioritized for screening over candidates with lower scores, but no filters or cutoffs were used to exclude orthologue candidates. Mammalian deafness genes with non-deafness associated paralogs were also considered in candidate identification.

### Fly strains and genetics

T2A-GAL4 and internally GFP or YFP tagged fly strains corresponding to fly genes that are orthologue candidates for human and mouse deafness genes were obtained from the Bloomington Drosophila Stock Center (https://bdsc.indiana.edu/), Kyoto Stock Center (https://kyotofly.kit.jp/cgi-bin/stocks/index.cgi), or from Dr. Hugo Bellen’s laboratory at Baylor College of Medicine (http://flypush.imgen.bcm.tmc.edu/lab/index.html). The full list of genotypes and stock numbers can be found in S1A and S1B Table. For gene expression studies, y w; UAS-CD8-GFP/CyO virgin females were crossed to T2A-GAL4 expressing males from MiMIC (Minos Mediated Integration Cassette) or CRIMIC (CRISPR Mediated Integration Cassette) lines [25] to express a membrane bound GFP in cell types that express the genes of interest. GFP-positive animals were visually inspected under a fluorescent microscope selected for dissection. When a visible GFP signal was not observed, at least 20 pupae were dissected and screened using confocal microscopy to identify a GFP signal in Johnston’s organ. For protein localization studies, internally GFP- or YFP-tagged protein expression lines from MiMIC [26, 27] or CTPI (Cambridge Protein Trap Insertion) collection [28, 29] were self-crossed. All flies were maintained at room temperature on standard food at 25°C. Loss of function mutants for Cad99C, Msp-300, and Koi were identified through Flybase (http://flybase.org/) and obtained from the Bloomington Drosophila Stock Center (https://bdsc.indiana.edu/). UAS-RNAi were used from the Transgenic RNAi Project (TriP) corresponding to each T2A-GAL4 gene. For GAL4/UAS based RNAi studies, female T2A-GAL4 flies were mated to male RNAi flies and F1 progeny were collected post-pupation.

### Immunolabeling, microscopy, and imaging

Fly tissues 24–48 hours post-puparium formation were dissected in PBS at room temperature and fixed with 4% paraformaldehyde in PBS for 20 minutes. Specimens were washed and permeabilized in 0.2% Triton X-100 in PBS three times for 10 minutes each. Standard protocols were followed for immunohistochemistry [24]. AlexaFluor 568 phalloidin (1:200, ThermoFisher Scientific, USA) was used to label F-actin to visualize scolopale cells. Samples were mounted in VECTASHIELD antifade mounting medium (Vector Laboratories, USA). Images were acquired using a LSM880 confocal microscope using a 63x objective lens (Zeiss, Germany) and visualized and processed using LSM Zen (Zeiss), and Photoshop software (Adobe, USA).

### Fly behavior studies

#### Housing and handling

All flies were grown in a temperature and humidity-controlled incubator at 25 °C and 50% humidity on a 12-hour light/dark cycle. Flies were reared on standard fly food (water, yeast, soy flour, cornmeal, agar, corn syrup, and propionic acid). Collection of socially naïve adults was performed by isolating pupae in 16x100 polystyrene vials containing approximately 1 ml of fly food. After eclosion, flies were anesthetized briefly with CO2 and examined to ensure they were healthy and lacking wing damage. Anesthetized flies were returned to their vials and allowed a full 24 hours to recover before testing.

#### Courtship assays

Courtship assays were performed in a 6 well acrylic plate with 16mm circular wells, with a depth of 1.5mm as described in [30, 31] with slight modifications. One control Canton-S male (aged 6–10 days), and one mutant virgin female (aged 6–10 days) were simultaneously introduced into the chamber via aspiration. The bottom of each well was covered by a fine mesh to allow for the playback of courtship sound while keeping the animal contained. The acrylic plate was placed directly on an output loudspeaker, with the mesh side facing the source of the artificial courtship song [32]. Courtship sound was played through the loudspeaker with an audio amplifier (Pyramid PA105 80W) plugged into a computer to play the sound file. Video recordings were captured using a digital (Basler 1920UM, 1.9MP, 165FPS, USB3 Monochromatic) camera using the BASLER Pylon module, with an adjusted capturer rate of 33FPS. Conversion of captured images into a movie file was performed via a custom MatLab script, and assessment of fly behavior was performed manually. The time to copulation was recorded using the in-software timestamp for reference. For the purposes of analysis, trials in which flies which were already copulating at the onset of filming were discarded.

### Electrophysiology

Electrophysiological recordings were performed with electrolytically sharpened tungsten electrodes inserted into the joint between the first and second antennal segments (recording electrode) and penetrating the head cuticle near the posterior orbital bristle (reference electrode), in response to near-field playback of computer-generated pulse song, as described in [33]. The signals were subtracted and amplified with a differential amplifier and digitized at 13 kHz. Sound evoked potentials (SEPs) were measured as the max-min values in the averaged trace from 10 consecutive presentations of the pulse song, as previously described.

### Fly cell atlas

The Fly Cell Atlas, a single-nucleus transcriptomic atlas of the adult fruit fly [34], was accessed using the Scope platform (https://scope.aertslab.org/#/FlyCellAtlas/). Specific genes were visualized using the 10x stringent setting of the whole antenna and images were taken for each expression profile. LogFC scores were obtained to assess specificity of genes to specific clusters. Flybase was accessed to perform a literature search for known JO expressing cell type genes using the anatomy/cell type search function. Lines from the scRNA-seq database screen were obtained from the Bloomington Drosophila Stock Center (https://bdsc.indiana.edu/), Kyoto Stock Center (https://kyotofly.kit.jp/cgi-bin/stocks/index.cgi).

## Results

### Many human and mouse deafness genes are conserved in the fruit fly

Large-scale, mutagenesis-based screens for genes involved in hearing have been conducted in Drosophila [35, 36] and mice [5–7] to identify candidates responsible for hearing loss. While the identification of these genes has been valuable to the scientific community, the biochemical functions of most of these genes remain poorly characterized. The time and cost of vertebrate model organism research still remains a major roadblock for throughput in discovery of underlying mechanistic function. The vast array of genetic tools available in Drosophila, its fast gestation time, and short life span all make the fruit fly an excellent system for functional testing of hearing related genes.

To identify orthologue candidates of human and mouse deafness genes in the fly, we used the DIOPT tool (version 7; [13]). Version 7 of DIOPT provides a ranking of orthologue candidate genes by interrogating a series of 15 orthologue prediction databases (Fig 1B). Genes with a higher DIOPT score typically have a higher degree of homology between genes in the compared species. We started with a list of already identified human deafness genes (hereditaryhearingloss.org) and mouse deafness genes recently identified by the International Mouse Phenotyping Consortium (https://www.mousephenotype.org/; [4]). Genes with the highest uncontested DIOPT score were identified as the primary orthologue candidate for this study. For genes that had multiple orthologue candidates tied for the highest score, each gene was treated as a potential orthologue candidate for screening purposes. To not rule out any potential orthologue candidates, no lower limit on DIOPT score was set as a cutoff. One gene, msp300, was added manually due to evolutionary conservation to members of the spectrin repeat containing nuclear envelope family, SYNE1 and SYNE2, being conserved in DIOPT but not the deafness gene SYNE4. Out of 169 human deafness genes, 134 had Drosophila candidates based on DIOPT (Table 1). Similarly, out of 67 mouse deafness genes, we were able to identify orthologue candidates for 52 of them (Table 2).

**Table 1:** List of orthologous human genes (DIOPT score >1) (DIOPT v7).

Deafness Locus/Syndrome	Human Gene Symbol	Drosophila Gene Symbol	DIOPT score (x/15)
DFNA1	*DIAPH1*	*dia*	8
DFNA2A	*KCNQ4*	*KCNQ*	7
DFNA4	*MYH14*	*zip*	8
DFNA6/14/38	*WFS1*	*wfs1*	10
DFNA7	*LMX1A*	*cg4328*	9
DFNA10	*EYA4*	*eya*	8
DFNA15	*POU4F3*	*acj6*	8
DFNA17	*MYH9*	*zip*	9
DFNA20/26	*ACTG1*	*Act5C*	8
DFNA20/26	*ACTG1*	*Act42A*	8
DFNA23	*SIX1*	*so*	9
DFNA25	*SLC17A8*	*VGlut*	9
DFNA27	*REST*	*cg9932*	2
DFNA28	*GRHL2*	*grh*	5
DFNA40	*CRYM*	*CG4872*	13
DFNA44	*CCDC50*	*CG10283*	3
DFNA51	*TJP2*	*pyd*	8
DFNA56	*TNC*	*CG8642*	3
DFNA56	*TNC*	*CG5550*	3
DFNA56	*TNC*	*CG31832*	3
DFNA56	*TNC*	*CG30280*	3
DFNA65	*TBC1D24*	*sky*	15
DFNA66	*CD164*	*vsg*	4
DFNA67	*OSBPL2*	*CG3860*	10
DFNA68	*HOMER2*	*homer*	10
DFNA70	*MCM2*	*Mcm2*	10
DFNA71	*DMXL2*	*Rbcn-3A*	10
DFNB2	*MYO7A*	*crinkled (ck)*	15
DFNB3	*MYO15A*	*Myo10A*	10
DFNB4	*SLC26A4*	*Prestin*	10
DFNB6	*TMIE*	*CG15130*	6
DFNB7/11-DFNA36	*TMC1*	*tmc*	5
DFNB8/10	*TMPRSS3*	*CG4613*	2
DFNB8/10	*TMPRSS3*	*CG3355*	2
DFNB9	*OTOF*	*misfire (mfr)*	11
DFNB12	*CDH23*	*ds*	3
DFNB12	*CDH23*	*cad87a*	2
DFNB12	*CDH23*	*cadN*	2
DFNB12	*CDH23*	*cad88c*	2
DFNB15/72/95	*GIPC3*	*kermit*	14
DFNB18	*USH1C*	*CG5921*	9
DFNB18B	*OTOG*	*Hml*	5
DFNB21-DFNA8/12	*Tecta*	*Hml*	3
DFNB23	*PCDH15*	*Cad99C*	9
DFNB24	*RDX*	*moe*	8
DFNB25	*GRXCR1*	*CG12206*	8
DFNB25	*GRXCR1*	*CG31559*	8
DFNB26	*GAB1*	*dos*	8
DFNB28	*TRIOBP*	*osp*	5
DFNB30	*Myo3a*	*ninaC*	12
DFNB31	*WHRN*	*dysc*	9
DFNB35	*ESRRB*	*ERR*	12
DFNB36	*ESPN*	*f*	4
DFNB37/DFNA22	*MYO6*	*jar*	12
DFNB39	*HGF*	*CG7432*	2
DFNB39	*HGF*	*Nrk*	2
DFNB44	*ADCY1*	*rut*	14
DFNB48	*CIB2*	*Cib2*	14
DFNB49	*BDP1*	*Bdp1*	8
DFNB49	*MARVELD2*	*Su(Tpl)*	3
DFNB57	*PDZD7*	*dysc*	3
DFNB60	*SLC22A4*	*Orct*	8
DFNB61	*SLC26A5*	*Prestin*	14
DFNB66	*DCDC2*	*DCX-EMAP*	2
DFNB66/67	*LHFPL5*	*Tmhs*	10
DFNB70	*PNPT1*	*PNPase*	14
DFNB74	*MSRB3*	*SelR*	13
DFNB76	*SYNE4*	*msp-300*	n/a
DFNB82	*GPSM2*	*pins*	13
DFNB84	*OTOGL*	*Hml*	3
DFNB84	*PTPRQ*	*PTP-ER*	2
DFNB84	*PTPRQ*	*Ptp10D*	2
DFNB84	*PTPRQ*	*Ptp4E*	2
DFNB84	*PTPRQ*	*CG42327*	2
DFNB86/DFNA65	*TBC1D24*	*sky*	15
DFNB88	*ELMOD3*	*CG10068*	2
DFNB89	*KARS*	*LysRS*	15
DFNB91	*SERPINB6*	*Spn55B*	9
DFNB93	*CABP2*	*CG30378*	2
DFNB93	*CABP2*	*CG13898*	2
DFNB94	*NARS2*	*AsnRS-m*	15
DFNB98	*TSPEAR*	*clos*	3
DFNB99	*TMEM132E*	*dtn*	13
DFNB100	*PPIP5K2*	*l(1)G0196*	14
DFNB101	*GRXCR2*	*CG12206*	4
DFNB101	*GRXCR2*	*CG31559*	4
DFNB102	*EPS8*	*aru*	8
DFNB103	*CLIC5*	*Clic*	9
DFNB105	*CDC14A*	*cdc14*	12
DFNB106	*EPS8L2*	*aru*	12
DFNB108	*ROR1*	*ror*	13
DFNX1	*PRPS1*	*CG6767*	11
DFNX2	*POU3F4*	*vvl*	9
DFNX5	*AIFM1*	*AIF*	9
DFNX6	*COL4A6*	*CG25C*	8
Alport Syndrome	*COL4A5*	*col4a1*	6
Alport Syndrome	*COL4A3*	*col4a1*	5
Alport Syndrome	*COL4A4*	*col4a1*	4
Brancio-Oto-Renal Syndrome	*EYA1*	*eya*	10
Brancio-Oto-Renal Syndrome	*SIX5*	*six4*	5
CHARGE Syndrome	*SEMA3E*	*sema2a*	3
CHARGE Syndrome	*SEMA3E*	*sema2b*	3
CHARGE Syndrome	*CHD7*	*kis*	11
Diabetes and Deafness, Maternally Inherited	*MTTK*	*dnk*	14
Jervell & Lange-Nielsen Syndrome 1	*KCNQ1*	*kcnq*	7
Pendred Syndrome	*KCNJ10*	*Irk1*	4
Pendred Syndrome	*KCNJ10*	*Irk2*	4
Pendred Syndrome	*FOXI1*	*fd19B*	3
Pendred Syndrome	*FOXI1*	*fd64A*	3
Perrault Syndrome 1	*HSD17B4*	*Mfe2*	15
Perrault Syndrome 2	*HARS2*	*HisRS*	12
Perrault Syndrome 3/DFNB81	*CLPP*	*ClpP*	15
Perrault Syndrome 4	*LARS2*	*LeuRS-m*	15
Perrault Syndrome 5	*TWNK*	*mtDNA-helicase*	13
Perrault Syndrome 6	*ERAL1*	*CG7488*	14
Stickler Syndrome 2	*COL11A1*	*vkg*	2
Treacher Collins Syndrome	*POLR1C*	*CG3756*	15
Treacher Collins Syndrome	*POLR1D*	*l(2)37CG*	15
Usher Syndrome 1G	*USH1G*	*Sans*	13
Usher Syndrome 3A	*CLRN1*	*CG14142*	8
Usher Syndrome 3B	*HARS*	*HisRS*	15
Waardenburg Syndrome 1	*PAX3*	*prd*	10
Waardenburg Syndrome 2A	*MITF*	*mitf*	11
Waardenburg Syndrome 4	*SOX10*	*sox100b*	6
Unclassified	*TRRAP*	*Nipped-A*	14
Unclassified	*SCD5*	*Desat1*	14
Unclassified	*SLC12A2*	*Ncc69*	14
Unclassified	*ELMOD1*	*CG10068*	12
Unclassified	*ESRP1*	*fus*	12
Unclassified	*PLS1*	*fim*	12
Unclassified	*WBP2*	*Wbp2*	11
Unclassified	*PDE1C*	*Pde1c*	9
Unclassified	*GRAP*	*drk*	7
Unclassified	*CLRN2*	*CG1103*	3
Unclassified	*IKZF2*	*CG12769*	3

Orthologues of identified human deafness genes listed with deafness locus/syndrome and DIOPT score (>1).

**Table 2:** List of orthologous mouse genes (DIOPT score >1) (DIOPT v7).

Mouse Gene Symbol	Drosophila Gene Symbol	DIOPT score (x/15)
*Aak1*	*Nak*	11
*Acsl4*	*Acsl*	11
*Acvr2a*	*put*	9
*Ankrd11*	*CG10984*	10
*Ap3m2*	*cm*	14
*Ap3s1*	*or*	12
*Atp2b1*	*PMCA*	8
*BAIAP2L2*	*IRSp53*	8
*Ccdc88c*	*Girdin*	12
*Cyb5r2*	*CG5946*	13
*Duoxa2*	*mol*	11
*Emb*	*Bsg*	12
*Eps8l1*	*aru*	6
*Eps8l1*	*CG8907*	6
*Ewsr1*	*caz*	7
*Gata2*	*grn*	12
*Gga1*	*Gga*	12
*Gpr50*	*moody*	5
*Gpr50*	*Tre1*	5
*Klc2*	*Klc*	7
*Klhl18*	*KLHL18*	13
*Med28*	*MED28*	12
*Mpdz*	*Patj*	7
*Myh1*	*Mhc*	7
*Nedd4L*	*Nedd4*	14
*Nek5*	*niki*	2
*Nek5*	*nek2*	2
*Nek5*	*png*	2
*Nfatc3*	*NFAT*	6
*Nin*	*Bsg25D*	7
*Nisch*	*CG11807*	10
*Nptn*	*Bsg*	6
*Odf3l2*	*CG10252*	12
*Orai1*	*olf186-F*	9
*Phf6*	*Phf7*	2
*Ppm1a*	*alph*	14
*Sema3f*	*Sema-2a*	4
*Slc4a10*	*Ndae1*	8
*slc5a5*	*CG10444*	5
*slc5a5*	*CG32669*	5
*Spns2*	*spin*	11
*Sun1*	*koi*	6
*Tmem145*	*CG9304*	6
*Tmem30b*	*CDC50*	5
*Tmtc4*	*CG5038*	15
*Tox*	*CG12104*	5
*Tram2*	*TRAM*	7
*Ube2b*	*Ubc6*	13
*Ube2g1*	*CG40045*	15
*Vti1a*	*Vti1a*	14
*Wdtc1*	*adp*	14
*Zcchc14*	*CG10492*	2
*Zcchc14*	*CG2709*	2
*Zcchc14*	*smg*	2
*Zcchc14*	*CG3800*	2
*Zcchc14*	*CG9715*	2
*Zfyve26*	*CG5270*	12

Orthologues of identified mouse deafness genes (identified through IMPC) listed with DIOPT score (>1).

### Many Drosophila orthologue candidates of mammalian deafness genes are expressed in Johnston’s organ

**Fig 2:**
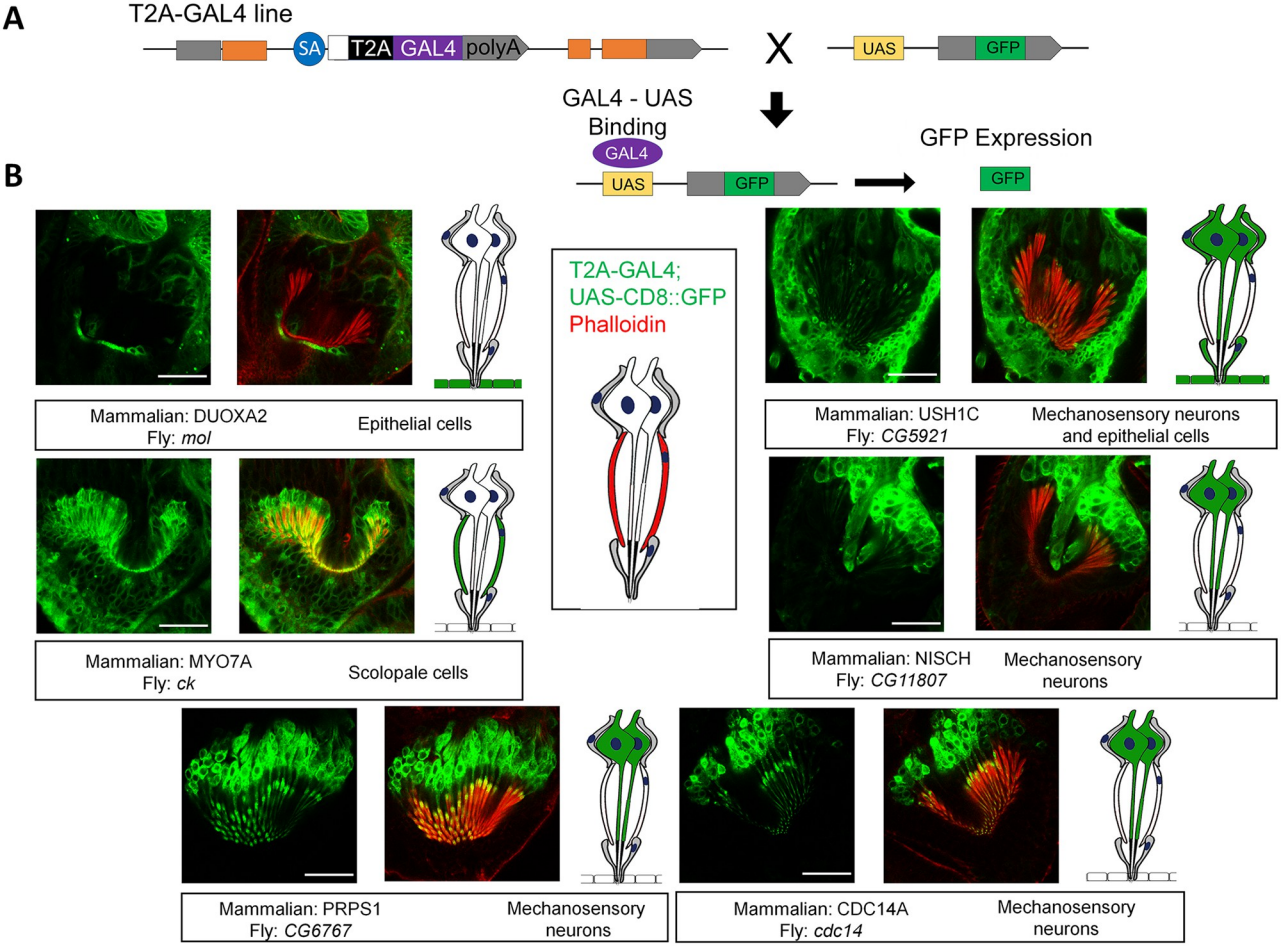
Johnston’s organ whole mount preparations showing GFP in the same temporal and spatial expression pattern as the deafness orthologue of interest. (A) Schematic showing progeny were generated from crossing GAL4 expressing flies to UAS-CD8 GFP flies. (B) Fluorescent images showing expression of individual genes. Fly and mammalian gene names are shown in boxes, as well as localization pattern description. A cartoon showing the expression pattern for each gene is shown to the side.

To test if the Drosophila orthologue candidates of mammalian deafness genes are expressed in the fly auditory organ, (Johnston’s organ) we used a collection of T2A-GAL4 gene trap cassette lines [25, 27, 37–40]. In these lines, a T2A-GAL4 cassette was inserted into the Drosophila genome, either randomly using recombination mediated cassette exchange (RMCE) of Minos-mediated transposons [26, 40] or through targeted CRISPR mediated insertion [25, 31]. These gene traps are located in an intron that flank two coding exons of a given gene, and the cassette encodes a splice acceptor sequence, an in-frame T2A ribosomal skipping peptide sequence and a GAL4 transcription factor coding sequence followed by a termination stop signal [37]. As a result, the N-terminus of the endogenous protein is transcribed and translated in these gene trap lines but is truncated due to the T2A-GAL4 sequence. At the same time, the GAL4 protein is translated in the same spatial and temporal expression pattern as the host gene (Fig 2A). When these flies are mated to a fly with a UAS sequence upstream of a fluorescent reporter (e.g. UAS-CD8::GFP, which expresses a membrane tagged GFP), one can assess the expression pattern of gene of interest [11]. We took advantage of this methodology and assessed whether the candidate Drosophila orthologues of human and mouse deafness genes were expressed in the Johnston’s organ, and if so, in which cell type.

Of the 39 gene trap lines that were screened (S2 Table), 26 lines were found to be expressed in Johnston’s organ (Table 3, Fig 2B). The fly genes that encode an orthologue of the Usher syndrome protein, MYO7A, and the MYH-9 related disease protein, MYO2A, were previously found to be localized at the distal tips of scolopale cells in Johnston’s organ using immunohistochemistry [24]. Our gene trap expression data confirmed that the candidate orthologues of both genes (MYO7A encoded by crinkled and MYO2A encoded by zipper in flies, respectively) are indeed expressed in scolopale cells. In addition, the Usher syndrome protein Harmonin (encoded by USH1C in human) encoded by the fly gene CG5921, and CDH23 protein, encoded by the fly gene dachsous, were expressed in the cap cell and epithelial cells near the attachment point of the a2/a3 junction. The fly genes cdc14 and CG6767, which correspond to the human deafness genes CDC14A and PRPS1 respectively, were both found to be expressed preferentially in Johnston’s organ mechanosensory neurons. CDC14A and PRPS1 are both known to be expressed in cochlear hair cells in mice [1, 41]. In addition to previously identified human deafness genes, orthologue candidates of several genes implicated in deafness in mice were also found to be expressed in Johnston’s organ. mol (Duoxa2) was expressed in epithelial cells near the attachment point of the scolopidia at the a2/a3 segment. CG11807 (Nisch) is expressed predominantly in mechanosensory neurons which suggests a role in neuronal function or development. Additional examples of genes screened by this method are shown in S1 Fig.

**Table 3:** Gene expression of human and mouse deafness orthologues using GFP overexpression.

Species discovered	Human gene	Fly orthologue candidate	DIOPT (x/15)	Gene Expression
mouse	*Aak1*	*Nak*	11	ubiquitious
mouse	*Acsl4*	*Acsl*	14	ubiquitious
human	*AIFM1*	*aif*	10	not detected
mouse	*Ankrd11*	*CG10984*	10	ubiquitious
mouse	*Atp2b1*	*PMCA*	13	ubiquitious
mouse	*Baiap2l2*	*Irsp53*	8	not detected
human	*CCDC50*	*cg10283*	6	ubiquitious
human	*CDC14A*	*cdc14*	12	mechanosensory neurons
human	*CDH23*	*ds*	3	attachment cells and mechanosensory neurons
human	*CHD7*	*Kis*	11	ubiquitious
mouse	*Col9a2*	*CG42342*	1	ubiquitious
human	*DMXL2*	*Rbcn-3a*	11	ubiquitious
mouse	*Duoxa2*	*mol*	11	epithelial cells
mouse	*Eps8l1*	*CG8907*	7	not detected
human	*GRHL2*	*grh*	7	attachment cells
human	*GRXCR1*	*CG31559*	9	not detected
human	*HOMER2*	*homer*	14	not detected
human	*HSD17B4*	*Mfe2*	15	not detected
mouse	*Il1r2*	*Toll-4*	1	mechanosensory neurons
mouse	*Myh1*	*Mhc*	11	not detected
human	*MYH14*	*zip*	11	ubiquitious
human	*MYH14*	*zip*	11	ubiquitious
human	*MYO15A*	*Myo10a*	10	scolopale cells
human	*MYO7A*	*ck*	15	scolopale cells
mouse	*Nedd4L*	*Nedd4*	14	ubiquitious
mouse	*Nisch*	*CG11807*	10	mechanosensory neurons
mouse	*Nptn*	*Bsg*	6	ubiquitious
mouse	*Odf3l2*	*CG8086*	12	mechanosensory and cap cells
human	*OSBPL2*	*cg3860*	13	neurons and cap cells
human	*PCDH15*	*Cad99c*	9	scolopale and attachment cells
human	*PNPT1*	*PNPase*	14	not detected
human	*POU4F3*	*acj6*	12	not detected
human	*PRPS1*	*Prps*	14	mechanosensory neurons
human	*RDX*	*Moe*	13	not detected
human	*SEMA3E*	*Sema2a*	3	ubiquitious
human	*SLC17A8*	*vglut*	10	ubiquitious
mouse	*Spns2*	*spin*	11	not detected
human	*TRIOBP*	*osp*	5	not detected
human	*TSPEAR*	*clos*	3	not detected
human	*USH1C*	*CG5921*	9	attachment cells and mechanosensory neurons

The Drosophila orthologues of human and mouse deafness genes with GFP expression patterns in Johnston’s organ whole mount preparations. GAL4 expressing transgenic flies for each deafness orthologue were used to drive GFP in the same spatial and temporal expression pattern for each gene. GFP expression in each cell type is noted for individual genes.

Next, we assessed the scRNA-seq data from the Fly Cell Atlas [34] to make comparisons between the expression data shown in our screen versus what had been identified from the single cell transcriptome analysis. By using this method, we can validate the gene expression from our screen by comparing the fluorescent expression patterns to the single cell clusters in the fly atlas. Of the known Johnston’s organ cell types, only the neuron of the scolopidia has been mapped to a specific cluster (Fig 4A, 4B). Since making these comparisons would be difficult with only one known cell type, we set out to identify the remaining JO cell type clusters. First, we performed a literature search using Flybase to identify genes known to be associated with the other scolopidia cell types. We then compared the sc-RNA seq data from these genes to see if we could identify a pattern in the clustering of these genes. Using this method, we were able to identify cluster candidates for the scolopale cell and ligament cell. However, we were not able to identify a candidate for the cap cell cluster. For the scolopale cell, the genes prospero and nervana two have been shown to be expressed in the chordotonal scolopale cells and have concentrated scRNA-seq expression to a specific cluster (Fig 4C; [42, 43]). For the ligament cell, six genes from the Flybase search had concentrated expression in a shared cluster and have been shown to be expressed in chordotonal ligament cells: pdm2, nub, repo, αTub85E, sr, and βTub56D (Fig 4D; [44–46]). To expand on the results of this preliminary screen, we tested 9 T2A-GAL4 lines of genes highly, and specifically, expressed in various unannotated clusters within the Fly Cell Atlas antennal data. We were not able to demonstrate a definitive cap cell or ligament cell cluster from this expression screen. In addition, we compared the results of our screen with the scRNA-seq database to further validate the results of our screen (Fig 4E). However, since the scRNA-seq database takes expression data from adult flies and our dissections were performed in the pupal stage; it is difficult to draw conclusions from discrepancies between the two data sets.

### The Drosophila orthologue candidates of many proteins encoded by mammalian deafness genes are also expressed and localized in Johnston’s organ

**Fig 3:**
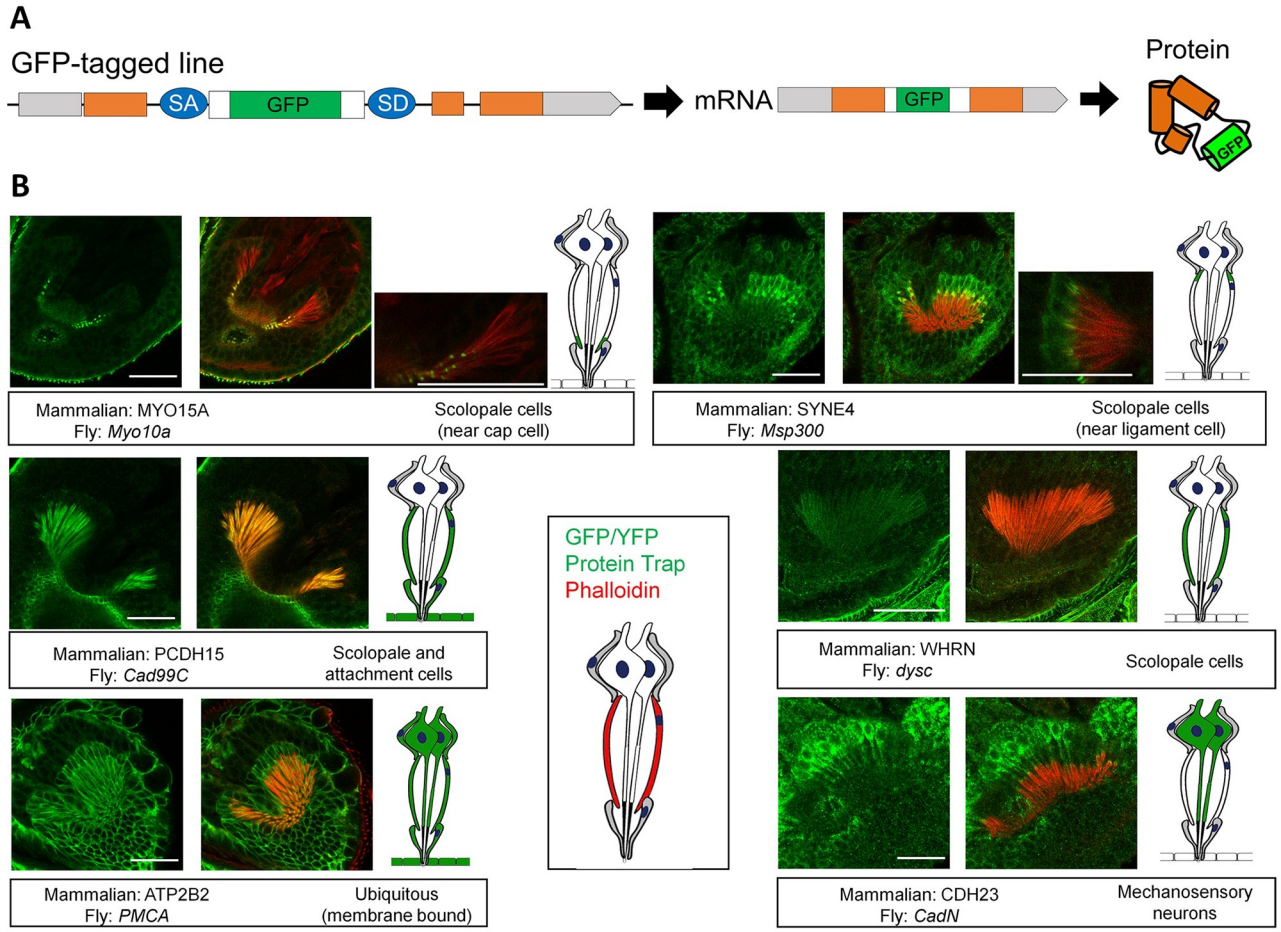
Johnston’s organ whole mount preparations showing GFP in the same temporal and spatial localization pattern as the deafness orthologue of interest. (A) Schematic showing progeny were generated from self-crossing GFP/YFP flies. (B) Fluorescent images showing expression of individual genes. Fly and mammalian gene names are shown in boxes, as well as localization pattern description. A cartoon showing the expression pattern for each gene is shown to the side.

While T2A-GAL4 gene trap lines are useful in identifying specific cell types in which a gene of interest is expressed, these tools are unable to provide information on where a given protein is localized within cells. We therefore used a collection of protein trap lines in which genes were internally tagged with a fluorescent reporter (GFP or YFP) separated from the native protein by a flexible linker sequence (Fig 3A). These strains were generated using RMCE of MiMIC insertions [26, 40] or through random integration of protein trapping piggyBac transposons [28, 29]. Although the protein tag is inserted in the middle of the protein rather than at its N- or C- termini, the majority (~75%) of these lines have been found to produce functional proteins [26].

Of the 30 protein trap lines that we screened (S3 Table), 26 lines showed expression in Johnston’s organ (Table 4; Fig 3B). In mammals, Myosin 15A protein localizes at the tips of hair cell stereocilia where it plays a role in elongation and maintenance of the hair bundle [47, 48]. The fly orthologue candidate of Myosin 15A, Myo10a, is localized to the distal tips of scolopale cells. This result was striking because scolopale cells and hair cell stereocilia seem to have conserved protein machinery, in addition to their abundant actin filaments [24]. In mammals, Myosin 15A also traffics a known Usher syndrome protein, Whirlin (encoded by dyschronic in flies) to the tips of hair cell stereocilia as a cargo protein. We found that fly Whirlin homologue was also expressed in scolopale cells along with the tip link proteins Protocadherin 15 (encoded by Cad99C in flies) and CDH23 (encoded by CadN in flies). ATP2B2 (encoded by PMCA in flies), which corresponds to a known genetic interactor of CDH23 that has shown to be implicated in deafness, was also localized ubiquitously throughout Johnston’s organ [49]. Another specific localization pattern observed was the deafness protein SYNE4 (encoded by msp300 in flies). Although SYNE4 was not identified as an orthologue candidate in DIOPT, likely due to low conservation, we included this in our screen because the SYNE family of protein are known to be homologous to Msp300 [50]. In mammals, SYNE4 is localized in the nucleus of outer hair cells where it plays a role in anchoring the nuclear membrane to the cytoskeleton of the cell through an interaction with another deafness protein, Sun1 (encoded. by koi in flies) [51, 52]. In flies, we found that Msp300 localizes to scolopale cells near the attachment of the ligament cell in a small, compartmentalized area. Although Sun1 and SYNE4 are known to localize in similar locations in mice within the cochlea, the localization pattern of their fly orthologue candidates in Johnston’s organ, while overlapping, is quite different. Koi localizes to the nuclear membrane of Johnston’s organ cell types arguing for a potential conserved role across species. Like our expression screen, several mouse deafness genes that have yet to be implicated in human deafness were found to be expressed in Johnston’s organ. Fly proteins corresponding to mouse Nptn, Slac4a10, and Nedd4L (encoded by the genes bsg, Ndae1, and Nedd4 in flies, respectively) all showed a ubiquitous expression in Johnston’s organ, suggesting they may also be involved in hearing in flies as well. Additional examples of genes screened by this method are shown in S2 Fig.

**Table 4:** Protein localization of human and mouse deafness orthologues using an endogenous GFP cassette.

Species discovered	Human gene symbol	Fly orthologue candidate	DIOPT (x/15)	Protein Localization	Cell Type(s)
mouse	*Acsl4*	*Acsl*	14	ubiquitous	ubiquitous
mouse	*ANKRD11*	*CG10984*	10	ubiquitous	ubiquitous
mouse	*Atp2b1*	*PMCA*	13	membrane bound	ubiquitous
human	*CD164*	*vsg*	6	cytoplasmic	ubiquitous
human	*CDH23*	*CadN*	2	cytoplasmic	mechanosensory neurons
human	*CHD7*	*kis*	11	nuclear	ubiquitous
human	*CLIC5*	*Clic*	10	cytoplasmic	ubiquitous
mouse	*COL9A2*	*CG42342*	1	ubiquitous	ubiquitous
human	*DCDC2*	*DCX-EMAP*	2	cytoplasmic (diffuse)	ubiquitous
human	*MARVELD2*	*Su(Tpl)*	3	nuclear	ubiquitous
human	*MSRB3*	*SelR*	13	not detected	not detected
mouse	*Myh1*	*Mhc*	11	ubiquitous	ubiquitous
human	*MYH14*	*zip*	11	ubiquitous	ubiquitous
human	*MYO15A*	*Myo10a*	10	distal tip at a2/a3 junction	scolopale cells
human	*MYO7A*	*ck*	15	cytoplasmic (concentrated at distal tip)	scolopale cells
mouse	*Nedd4L*	*Nedd4*	14	ubiquitous	ubiquitous
mouse	*Nptn*	*bsg*	6	ubiquitous	ubiquitous
human	*PCDH15*	*Cad99c*	9	co-localizes with actin in scolopale cells and ubiquitous in epithelial cells	scolopale/epithelial cells
human	*PRPS1*	*CG6767*	14	nuclear	mechanosensory neurons
human	*PTPRQ*	*Ptp4E*	2	nuclear	mechanosensory neurons
human	*PTPRQ*	*Ptp10D*	2	cytoplasmic (co-localizes with actin)	scolopale cells
human	*SEMA3E*	*Sema2a*	3	ubiquitous	ubiquitous
human	*SLC17A8*	*vglut*	10	ubiquitous	ubiquitous
mouse	*Slc4a10*	*Ndae1*	12	ubiquitous	ubiquitous
human	*SUN1*	*Koi*	6	nuclear membrane	ubiquitous
human	*SYNE4*	*Msp-300*	n/a	ubiquitous (concentrated at proximal tip)	scolopale cells
human	*TJP2*	*pyd*	10	membrane bound	epithelial cells
human	*TMC1*	*Tmc*	5	not detected	not detected
human	*TRIOBP*	*osp*	5	not detected	not detected
human	*WHRN*	*dysc*	9	ubiquitous	scolopale cells

The *Drosophila* orthologues of human and mouse deafness genes with fluorescent protein localization in Johnston’s organ whole mount preparations. Subcellular localization in Scolopidia cell types and protein localization in each cell type is noted for individual genes.

**Fig 4:**
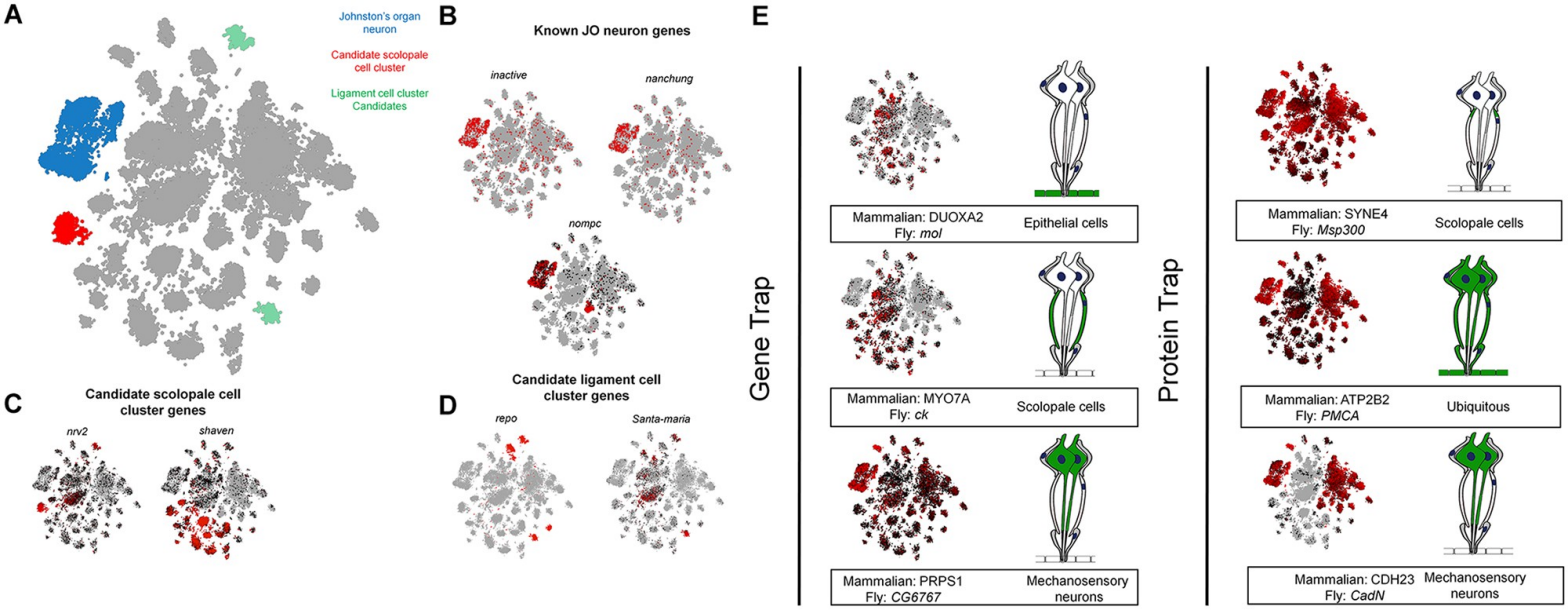
(A) scRNA-seq data for the antennal cell cluster showing known and candidate clusters of specific cell types. (B) scRNA-seq data for known Johnston’s organ neuron genes. (C) scRNA-seq data for candidate Johnston’s scolopale cell cluster genes. (D) scRNA-seq data for candidate Johnston’s ligament cell cluster genes. (E) Gene trap and protein trap lines from theexpression/localization screen showing corresponding scRNA-seq data.

Like the gene expression screen, we investigated the data from the Fly Cell Atlas [34] to compare information from scRNAseq experiments to data and proteins identified to be expressed in the fly Johnston’s organ based on our screen. Like the expression screen shown previously, we tested 3 additional YFP positive lines to attempt to determine the ligament and cap cell clusters. However, were not able to narrow down either cell type to a specific cluster (Fig 4).

### Behavioral and electrophysiological based functional assessment of selected genes

**Fig 5:**
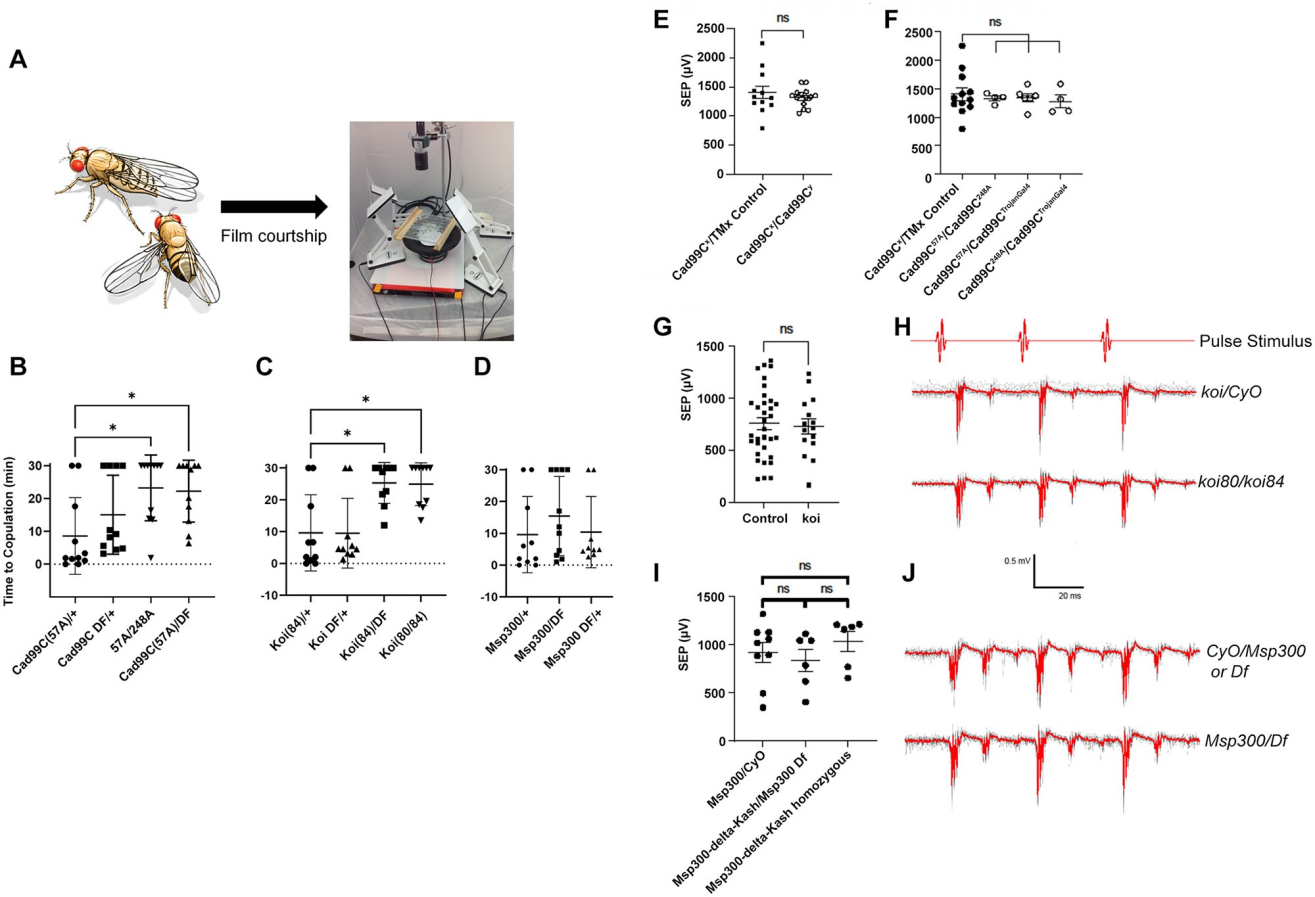
(A) Depiction of electrophysiology rig for testing sound-evoked potentials (SEPs). (B-D) Time to copulation for experimental and control conditions for the genes *Cad99C*, *koi*, and *msp300* respectively. A wild type male was placed in a video recording chamber with a female of the relevant genotype and the time taken for copulation measured from a playback of the recording. If no copulation was observed by 30 minutes, the trial was halted. An asterisk denotes a p-value less than 0.05 for all experiments (Mann–Whitney U test) (E-J) Sound evoked potentials (SEPs) showing voltage recorded for experimental and control groups for the genes *Cad99C*, *koi*, and *msp300* respectively. Sample traces are shown for *koi*, and *msp300*. ns = not significant.

Considering that fly orthologue candidates of many mammalian hearing impairment genes were found to be expressed in the Johnston’s organ, we explored the functional relevance of a subset of these genes in fly hearing. To accomplish this, we employed both behavioral and electrophysiological approaches to directly test whether these genes are necessary for hearing. Our behavioral approach took advantage of the Drosophila courtship song produced by males received by females. This courtship song is one of the key determinants for a female’s response to copulation attempts from the male. The absence of a courtship song or a defect in the female’s ability to hear the song results in an increase in time to copulation or complete rejection of courtship advances by the male [53, 54] (Fig 5A). Taking advantage of this paradigm, we obtained or generated genetically manipulated virgin female flies and housed them in single-pair matings with wild-type (Canton-S) male flies. Using a high-speed camera, we quantified the time from the start of courtship until a successful copulation occurs, up to 30 minutes when the experiment was halted. For this assay, we selected three genes (Cad99C, koi and Msp300) in which a null or strong loss-of-function allele has been established and characterized to be homozygous or hemizygous viable [55, 56]. Two of the three genes tested using mutant alleles crossed to deficiencies, Cad99C and koi, had a statistically significant delayed time to onset of courtship (Fig 5B–5D). In addition, Msp300 mutant flies appeared to show a bimodal distribution where roughly half of the flies tested failed to copulate and the other half mated normally compared to the wild type.

To assess if our Drosophila mutants had hearing defects, we recorded sound-evoked compound action potentials from the antennal nerve ([24, 57–59]; Fig 5E). We tested null alleles of our three hemizygous or homozygous viable mutant lines tested the courtship assay: Cad99C, Msp300, and koi. We tested mutant/deficiency or trans-heterozygotes mutant combinations and found no significant difference in SEPs compared to their controls (Fig 5F–5K). For Cad99C, we previously showed that loss of function of this gene cause only very mild morphological defects in Johnston’s organ [24], which may explain the lack of an obvious hearing defect in these mutants. Together, these suggest that the behavioral defects seen in loss of function mutants in these three genes are due to factors independent of hearing function, or minor hearing defects that is not captured by SEP could contribute to this defect.

### Using a T2A-GAL4/RNAi approach to further test the role of conserved mammalian deafness genes in fly mating and hearing

**Fig 6:**
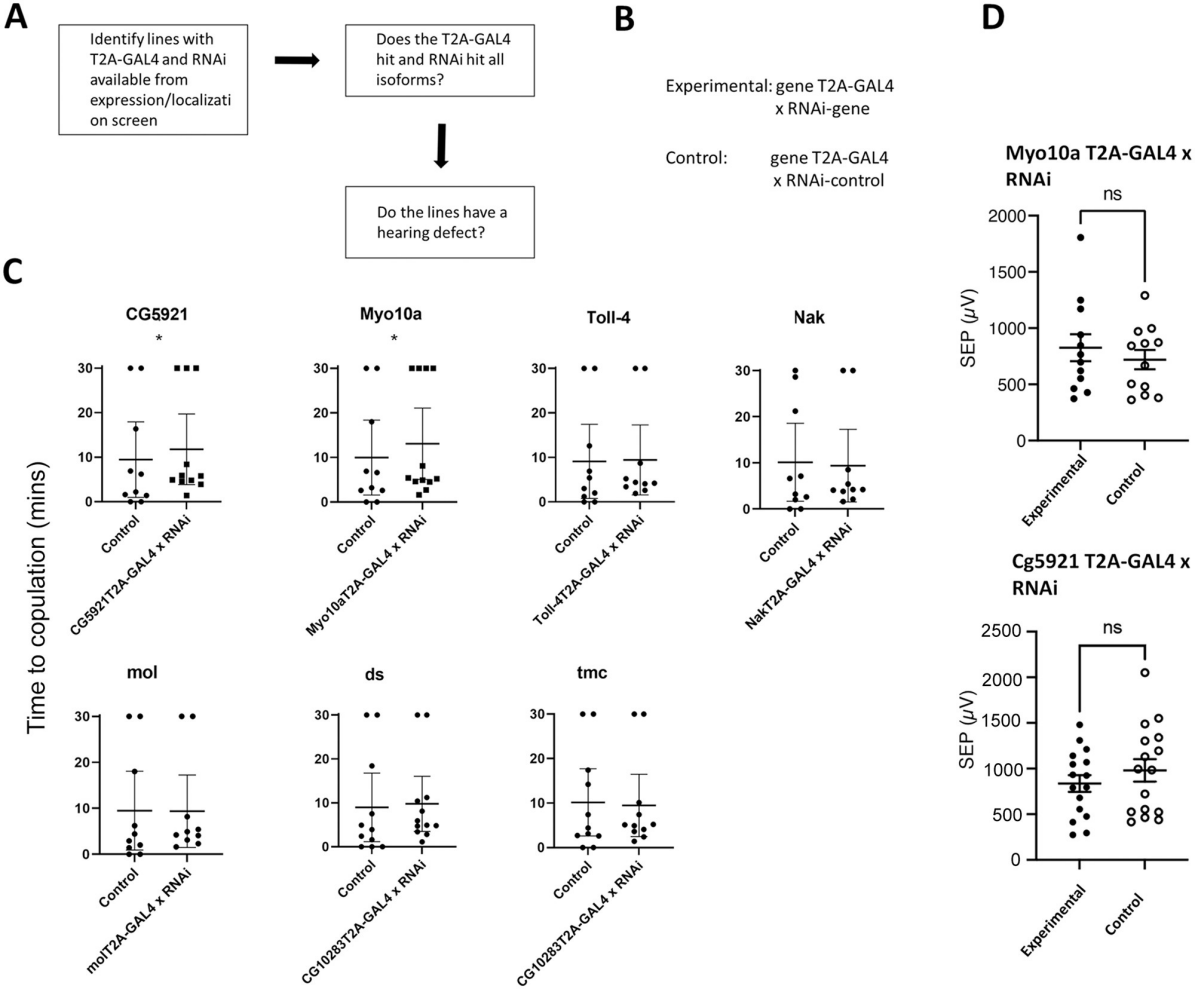
(A-B) Experimental pipeline for testing T2A-GAL4 x RNAi experimental and control conditions. (C) Time to copulation for experimental and control conditions for each gene tested. An asterisk denotes a p-value less than 0.05 for all experiments (Mann–Whitney U test) (D) Sound evoked potentials of experimental and control groups for the genes myo10a and cg5921. An asterisk denotes a p-value less than 0.05 for all experiments (Mann–Whitney U test).

One limitation to investigating the function of a gene in hearing is that some genes are essential, prohibiting us from conducting behavioral assays and electrophysiological recordings on strong loss-of-function alleles. To expand the results of our previous screen and increase the number of genes that we could test in adult flies through our screening paradigms, we employed a gene knockdown approach using T2A-GAL4 gene trap lines mated to UAS-RNAi lines to knock down, but not eliminate, expression of our genes of interest (Fig 6A and 6B). Using this approach, we estimated that we could increase the number of loss-of-function mutants that were viable in the adult for behavioral and SEP testing. We identified eight genes out of 18 that were viable in the adult using RNAi’s. Two of these genes, Myo10a (which correspond to human MYO15A) and CG5921 (homologous to human USHC1C), had statistically significant increased times to copulation using our behavioral testing paradigm (Fig 6C). However, neither of these two genes had increased SEPs (Fig 6D) suggesting the behavioral difference is due to factors independent of hearing.

## Discussion

Conservation of gene function between Drosophila and mammals was previously explored to identify molecular similarities between hearing organs [36]. Functional conservation of hearing mechanisms was also investigated for a small number of genes [24, 58, 59]. However, no comprehensive screen identifying orthologue candidates of known mammalian deafness genes in flies and assessing their expression in the fly Johnston’s organ had been performed until this study. This was the first attempt at a comprehensive screen in the fruit fly using known mammalian deafness orthologues. Our goal was to increase the list of known human and mouse deafness genes conserved in Drosophila and expressed in the Johnston’s organ of to more easily study the molecular and cellular functions of the proteins coded by these genes in a tractable and rapid model system.

We found that most human and mouse deafness genes had at least one identifiable Drosophila orthologue candidate using DIOPT. Of the 152 mammalian genes we screened, a large number (n = 145) had an identifiable DIOPT score which suggests potential conservation. In addition, some of the highest rated fly orthologous candidate genes using DIOPT scores, such as crinkled (orthologous to human MYO7A, DIOPT Score = 15/15) and Cad99C (orthologous to human PCDH15, DIOPT Score = 11/15) were previously found to be functionally relevant to fly hearing [24, 58, 59]. This suggests that the degree of conservation using DIOPT may be used as a predictor of functional conservation and could be potentially used in the future as a means of prioritizing genes for functional analysis in Drosophila.

We noted some of the genes with known localization and/or expression patterns within the mammalian organ of Corti had interesting expression patterns within Johnston’s organ scolopidia suggesting functional similarity. The fly orthologue candidates of Usher syndrome genes MYO7A (crinkled in fly), WHRN (dyschonic in fly), USH1C (CG5921 in fly), and PCDH15 (Cad99C in fly) were localized or expressed in the actin-rich scolopale cells, consistent with our previous findings [24]. In mammals, Usher syndrome proteins are localized to the actin-rich stereocilia of hair cells. Although these proteins are localized in different cell types in flies and mammals, their congregation together in an F-actin rich environment argues for functional homology [23]. We also found that the protein encoded by the fly orthologue candidate of MYO15 (myo10a in fly), which is normally localized at the distal tips of hair cell stereocilia in mammals, was localized to the distal tips of scolopale cells. Mammalian MYO15 plays a role in formation and maintenance of hair cell stereocilia bundles by carrying cargo, including the Usher syndrome protein WHRN (fly: Dysc), to the tips of stereocilia rows [47, 48, 60–62]. Myo10a in fruit flies may be playing a similar role by elongating the scolopale cells during development and/or maintaining the structural integrity of the stretch receptor throughout adulthood by carrying cargo proteins to the distal tips.

We used a behavioral paradigm that allows quick and efficient interrogation of the functional characteristics of mutants in three conserved deafness genes in the fruit fly: Msp300, koi, and Cad99C. Cad99C is homologous to the Usher syndrome gene PCDH15 while Msp300 and koi correspond to the nuclear membrane anchoring genes SYNE4 and SUN1, respectively. Like other conserved Usher syndrome proteins, Cad99C localizes to scolopale cells near the attachment point at the a2/a3 antennal joint of Johnston’s organ where it is believed to play a role in anchoring the scolopidia to the cuticle [24]. Our behavioral paradigm showed a statistically significant increase in the time to copulation in mating a wild-type male fly with a female with a mutant allele of Cad99C. This suggests the absence of functional Cad99C causes a phenotypic effect consistent with hearing loss in females. In addition, Koi deficient females also showed a delay in copulation time but not in Msp300 mutant flies. In mice, Sun1 and Nesprin 4 (protein encoded by the SYNE4 gene) are localized to the nuclear membrane of outer hair cells where they play a role in anchoring to the cytoskeleton [51, 52]. In flies, the SUN1 homologue (Koi) but not the SYNE4 homologue (Msp300) localizes to the nuclear membrane of Johnston’s organ cell types. Interestingly, our Msp300 mutant females displayed a bimodal distribution where roughly half of the flies tested had normal copulation times and the other half had significantly delayed copulation times. This could be due to incomplete penetrance of this phenotype or a sensitivity phenotype where exposure to environmental factors significantly worsen this behavioral outcome. In the future, it would be interesting to explore this relationship by exposing Msp300-mutant flies to loud noise before behavioral testing. We also tested the female mating responses on eight genes in which we obtained viable adult flies from T2A-GAL4/UAS-RNAi mediated knockdown. Two of these genes, Myo10a and CG5921, had statistically significant increased times to copulation. The mammalian orthologue of the fly gene Myo10a, Myo15a, is known to traffic cargo to the tips of hair cell stereocilia and is implicated in maintenance and development of the stereocilia bundle [63]. Usherin, encoded by the fly gene CG5921, is implicated in usher syndrome type 1, an autosomal recessive sensory disorder that causes deafness, blindness, and vestibular defects [64].

Our electrophysiological testing allowed us to test if there is a defect in the hearing response. We analyzed the null or strong loss of function alleles of the three non-essential genes tested in the behavioral screen as well as knockdown of two additional genes with behavioral phenotypes using the T2A-GAL4/UAS-RNAi method and found no significant difference in SEP’s. This suggests that the behavioral defects are due to factors independent of hearing function. However, the lack of an electrophysiological phenotype could also be caused by the challenge of achieving sufficient knockdown of the gene of interest to cause a phenotype and achieving too much knockdown and causing lethality. Alternative methods of auditory testing, such as noise exposure and age-related assays, may reveal differences in hearing in future studies. In addition, screening methods using electrophysiological testing would likely be more conducive to finding hearing defects in the fly. Gravitaxis testing would also be an effective method of screening as Johnston’s organ is known to function in wind direction and gravity sensing [65].

The molecular and mechanistic understanding of many of the identified mammalian deafness genes have not yet been characterized while others remain poorly characterized. While this work did not find novel fly deafness genes, the potential for Drosophila to rapidly study human hereditary deafness genes is very promising and provides several key opportunities. The Undiagnosed Disease Network has shown that model organism research, particularly in Drosophila, can provide important mechanistic insight into the function of individual genes and can lead to the development of novel therapies in humans. Despite the anatomical differences between the fly and human hearing organs, the significant genetic homology between hearing-related genes in human and fly combined with the genetic toolkits available make Drosophila a great system for assaying both candidate mammalian deafness genes and gene variants, including human pathogenic variants, in a high-throughput manner. Using these tools, one can probe into the mechanisms by which these proteins act and understand how pathogenic variants disrupt protein function. The insights into protein function and pathogenic mechanisms gained through using the fruit fly to study human deafness may ultimately lead to the development of novel therapies and less invasive treatments, as it has in other genetic diseases.

## Supporting information

S1 Fig Johnston’s organ whole mount preparations showing GFP in the same temporal and spatial expression pattern as the deafness orthologue of interest and sc-RNA seq data of deafness orthologues from the fly cell atlas database. Progeny were generated from crossing GAL4 expressing flies to UAS-CD8 GFP flies.
(TIF)

S2 Fig Johnston’s organ whole mount preparations showing localization of GFP-tagged proteins of deafness orthologues and sc-RNA seq data of deafness orthologues from the fly cell atlas database. (TIF)

S1 Table A complete list of orthologous human and mouse genes tested in this study. Orthologues of identified human and mouse deafness genes (identified through IMPC) listed with DIOPT score, flybase ID, and known GFP/YFP or T2A-GAL4 insertion transgenic fly lines.
(XLSX)

S2 Table Gene expression of human and mouse deafness orthologues using GFP reporters. The Drosophila orthologs of human and mouse deafness genes with GFP expression patterns in Johnston’s organ whole mount preparations. GAL4 expressing transgenic flies for each deafness ortholog were used to drive GFP in the same spatial and temporal expression pattern for each gene. GFP expression in each cell type, flybase ID, transgenic fly stock number, and genotype is noted for individual genes.
(XLSX)

S3 Table Protein localization of human and mouse deafness orthologues using an endogenous GFP cassette. The Drosophila orthologs of human and mouse deafness genes with fluorescent protein localization in Johnston’s organ whole mount preparations. Subcellular localaztion in Scolopidia cell types and protein localization in each cell type, flybase ID, genotype, and transgenic fly stock number is noted for individual genes.
(XLSX)
