# Supplementary figures and images for: Comparative exploration of mammalian deafness gene homologues in the Drosophila auditory organ shows genetic correlation between insect and vertebrate hearing
Source: PLoS One. 2024 Feb 27;19(2):e0297846. doi: 10.1371/journal.pone.0297846 (PMC10898740; doi:10.1371/journal.pone.0297846)

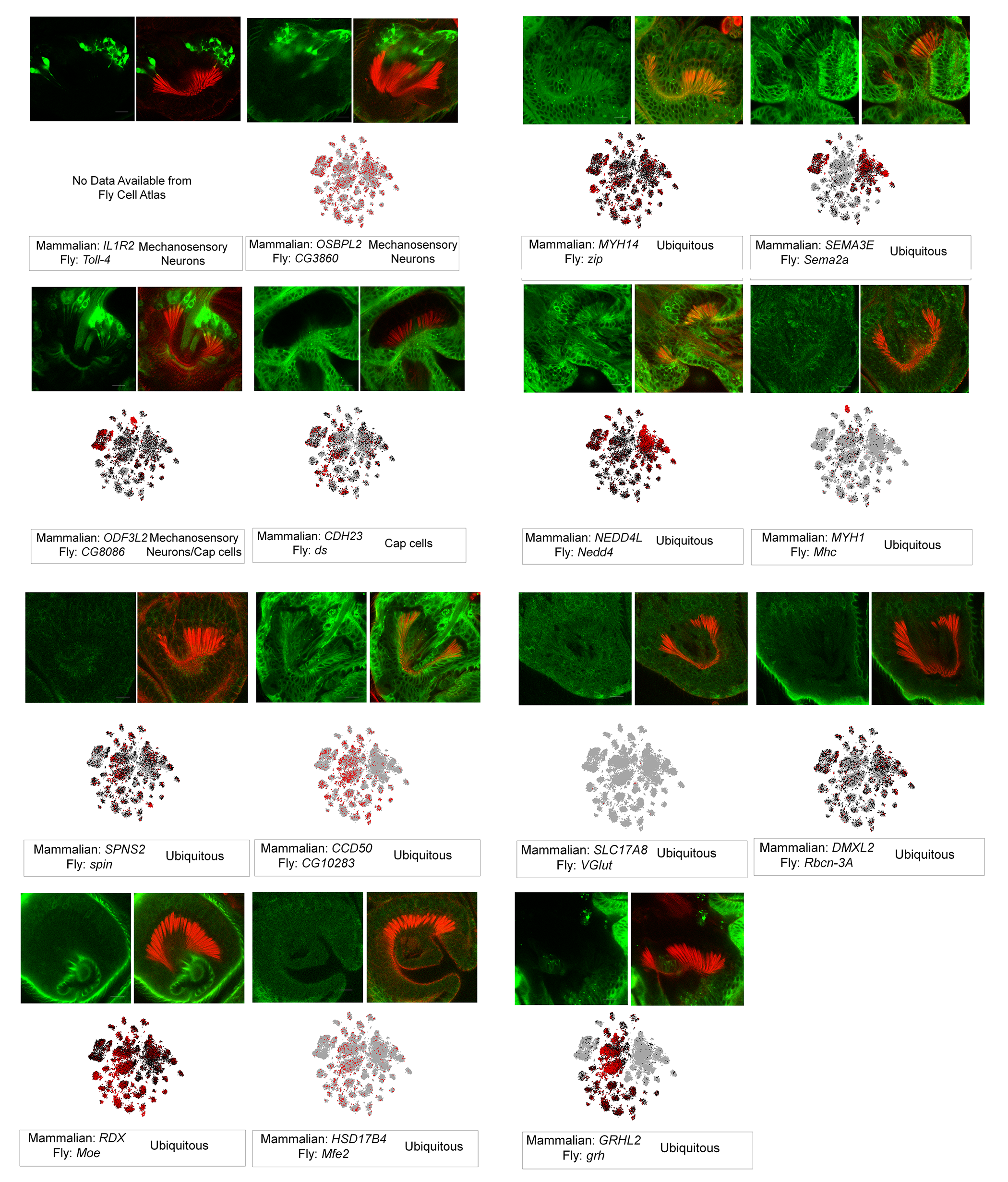

Supplement: S1 Fig — Progeny were generated from crossing GAL4 expressing flies to UAS-CD8 GFP flies. (TIF) [file pone.0297846.s001.tif]

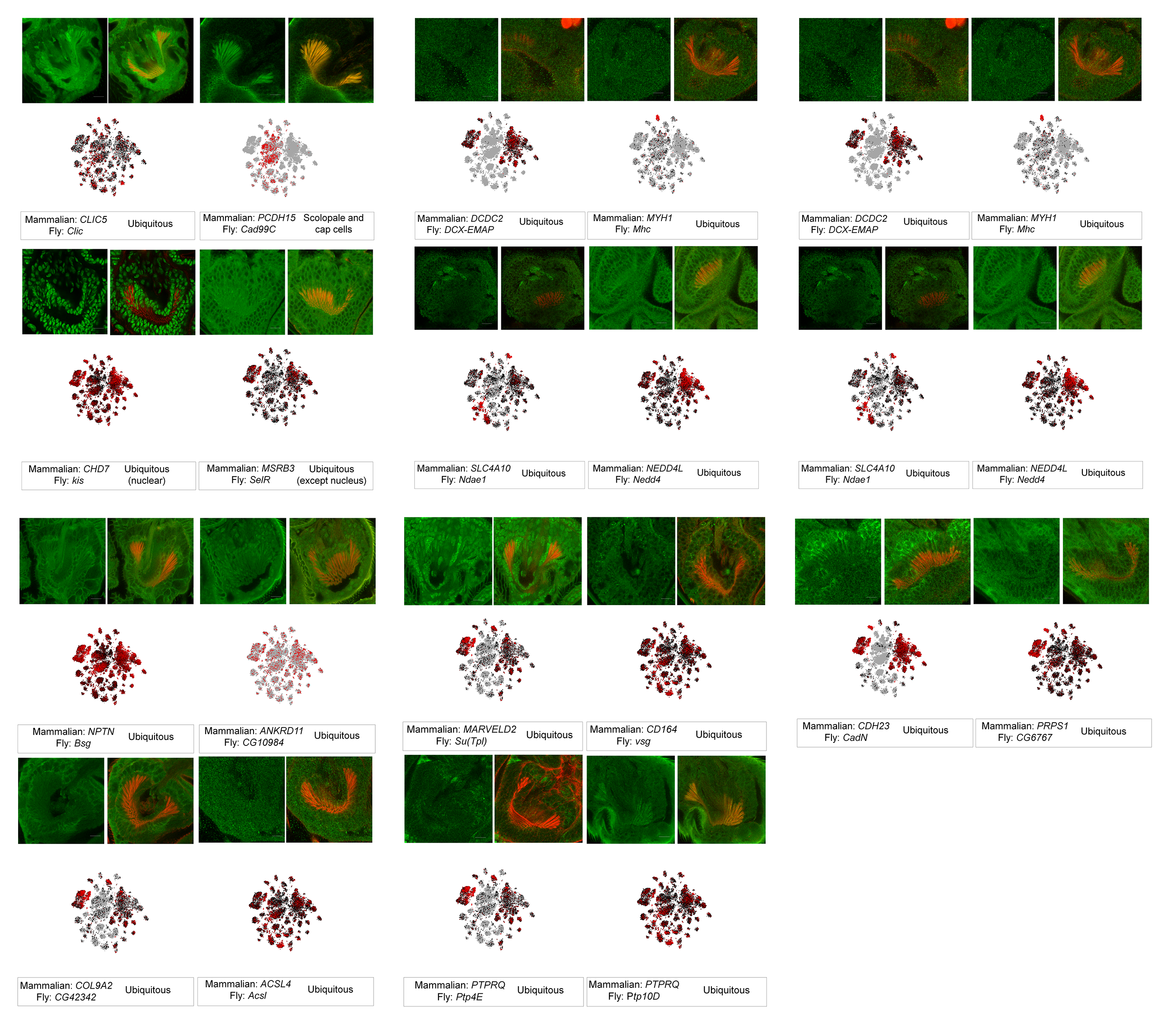

Supplement: S2 Fig — (TIF) [file pone.0297846.s002.tif]
